# Comparing Three Types of Mandarin Powders Prepared via Microfluidic-Jet Spray Drying: Physical Properties, Phenolic Retention and Volatile Profiling

**DOI:** 10.3390/foods10010123

**Published:** 2021-01-08

**Authors:** Xiao Chen, Joanna Le Hoong Ting, Yaoyao Peng, Pipat Tangjaidee, Yongchao Zhu, Qili Li, Yang Shan, Siew Young Quek

**Affiliations:** 1 Food Science Programme, School of Chemical Sciences, The University of Auckland, Auckland 1010, New Zealand; xche622@aucklanduni.ac.nz (X.C.); joannating38@gmail.com (J.L.H.T.); ypen083@aucklanduni.ac.nz (Y.P.); ptan226@aucklanduni.ac.nz (P.T.); yzhu313@aucklanduni.ac.nz (Y.Z.); 2 Hunan Agricultural Product Processing Institute, Hunan Academy of Agricultural Sciences, Changsha 410125, China; liqili626@163.com; 3 Hunan Province International Joint Lab on Fruits & Vegetables Processing, Quality and Safety, Changsha 410125, China; 4 Riddet Institute, Centre of Research Excellence in Food Research, Palmerston North 4474, New Zealand

**Keywords:** mandarin, microencapsulation, spray drying, phenolic compound, aroma compound, fruit powder, PCA

## Abstract

This study aimed to offer an alternative way for delivering the benefits of the mandarin fruit juice to consumers via spray drying microencapsulation. Two mandarin cultivars, Afourer (A) and Richard Special (RS), were studied. Three types of juice sample were prepared, i.e., the whole fruit juice (A3 & RS3), the flavedo-removed fruit juice (A2 & RS2), and the peel-removed fruit juice (A1 & RS1) samples. Gum Acacia and maltodextrin (ratio of 1:1, *w*/*w*) were chosen as wall matrices for aiding the drying of the juice samples while using a microfluidic-jet spray dryer. The properties of the fruit powder (colour, water activity, bulk/trapped density, solubility, hygroscopicity, morphology) and the retention of major phytochemicals (i.e., phenolic and volatile compounds) were examined. The results showed that the powders produced from the whole fruit juices (A3 and RS3) gave higher yellow colour with a regular winkled surface than other powders (A1 & RS1, and A2 & RS2). The water activity of mandarin powders was in a range of 0.14 to 0.25, and the solubility was around 74% with no significant difference among all of the powders. The whole fruit powders had a significantly higher concentration of phenolic compounds (A3, 1023 µg/100 mg vs. A2, 809 µg/100 mg vs. A1, 653 µg/100 mg) and aroma compounds (A3, 775,558 µg/L vs. A2, 125,617 µg/L vs. A1, 12,590 µg/L). This study contributed to the delivery of phenolic and flavour compounds of the mandarin fruits, at the same time minimising waste generation during processing. It also gave insight into the production of spray-dried powders from the whole mandarin fruits.

## 1. Introduction

Citrus fruits are members of the *Rutaceae* family, comprising 1600 species and 150 genera [1]. They are important horticultural crops that contribute to human diets globally [1,2]. Mandarin is a juicy fruit with a globular to oblate shape and deep- to reddish-orange skin, representing an easy peeling group of citrus variety.

Mandarin fruit consists of peel (pericarp) and pulp (endocarp). The endocarp is the flesh of mandarin fruits, typically containing 3–7 seeds and nine to 14 easily detached segments [3]. The peel or pericarp can be divided into an outer, orange coloured part, called exocarp or flavedo, and the inner colourless part, which is called mesocarp or albedo (Figure S1). Some of the components contained in the exocarp, such as paraffin waxes, essential oils, fatty acids, and pigments, have a critical role in protecting the fruits against insects and excessive sunlight exposure [1]. These compounds, especially the phenolic acids and flavonoids, also show diverse health benefits, including anticancer [4,5], antioxidant [4,6,7], anti-inflammatory [4], and antibacterial activities [4,7,8]. Mandarin peel is a rich source of essential oil and pectin, which are used in pharmaceutical and food industries to deliver substantial economic value [9,10,11]. However, the peel is normally discarded as a by-product during fruit consumption and juice processing.

Mandarins are strongly characterized by their pleasant flavour and high nutritional values and, therefore, have been well accepted by consumers. Mandarins have been manufactured into a wide range of products, including natural juices, jellies, candies, and syrup [1], in addition to its consumption as fresh fruit. Recently, there is a growing interest in the development of healthy functional foods while using natural substances. A number of research have been conducted to fortify food products with mandarin-derived bioactive components as the consumer trend moves toward the use of fruit in functional food products. For example, mandarin peel was added in biscuits dough in order to improve the nutritive value and strengthen the antioxidant properties [12]; mandarin low pulp juice has been successfully incorporated into an apple snack by vacuum impregnation technology [13]. 

Nowadays, functional ingredients in a dry format are in better market demand. Removing the water content in fruits could reduce quality loss that is induced by enzymatic hydrolysis and microbial growth while ensuring shelf-life stability during storage and transportation [14]. Furthermore, this will make the fruit product available all year round, overcoming the issue of seasonality. For mandarin fruits, they are more perishable than other citrus varieties, as their sensorial acceptability deteriorates quickly after fruit harvesting [1]. However, making fresh mandarins into powder forms could be an effective way to maintain their nutritional and sensorial values.

**Figure 1:**
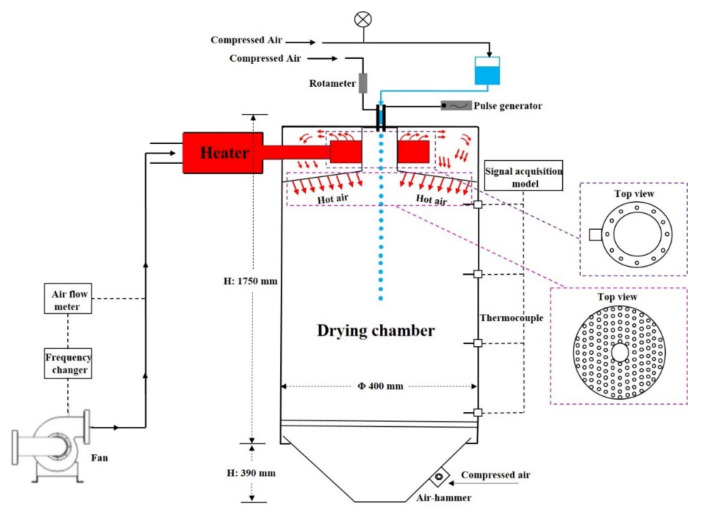
Schematic diagram of the microfluidic-jet (MFJ) spray dryer.

On the other hand, spray drying has long been used as an efficient way to produce powders from fruits [15,16]. It has many advantages, including the ease of operation and scale up for commercialisation, which offer lower cost and higher throughput when ompared to other drying techniques. Moreover, it produces powders with good flowability and stability [17]. In the last few decades, advancement in the spray drying technique has enabled the application of this method into a broad range of industries. The microfluidic-jet (MFJ) spray drying, as illustrated in Figure 1, is one of the successful attempts made by Wu et al. in 2007 [18], aiming to manufacture particles with a uniform size with identical morphology. To date, the spray drying of mandarin juices has been reported [19,20], but the application of MFJ spray drying technique has obviously not been explored. Furthermore, it is noted that previous studies have been focused on either the flavour or nutrient profile of the mandarin pulp juices during processing, or the utilisation of mandarin peels as a source of bioactive ingredients. No study has investigated the feasibility of using whole mandarin fruit as a functional ingredient or trying to transform the whole fruit into a product.

The main objective of this study was to test the concept of using the whole fruit juices in order to produce mandarin fruit powders via MSJ spray drying, by examining the physical properties, phenolic retentions, and volatile profiles of the produced powders. Spray drying microencapsulation of mandarin juices from different sample preparation, i.e., whole fruit, flavedo-removed, and peel-removed juice samples, were compared in order to achieve the objective. To the best of our knowledge, this approach is novel and has not been attempted. The approach offers an alternative way of delivering the health benefits of mandarin fruits to consumers while filling the knowledge gap in the production of spray-dried whole mandarin fruit powders.

## 2. Materials and Methods

### 2.1. Materials and Chemicals

Two cultivars of mandarin, Afourer (A) and Richard Special (RS), were kindly donated by the First Fresh (NZ) Ltd. (Gisborne, New Zealand). Two carrier materials for spray drying, maltodextrin (MD: 10–13 DE) and gum arabic (GA), were purchased from Ingredion Ltd. (Singapore) and Hawkins Watts Ltd. (Auckland, New Zealand), respectively. The chemicals were sources, as below: 2,4,6-Tripyridyl-s-Triazine (TPTZ), gallic acid, and 6-hydroxy-2,5,7,8-tetramethylchroman-2-carboxylic acid (Trolox), were purchased from Sigma–Aldrich (Auckland, New Zealand); 2,2-azino-bis-3-ethylbenzothiazaoline-6-sulfonic acid (ABTS) was purchased from Abcam Inc. (Melbourne, VIC, Australia); formic acid was obtained from EMD Millipore Corporation (Billerica, MA, USA). 

Authentic standards, including chlorogenic acid (≥95%), ferulic acid (≥99%), p-coumaric acid (≥98%), vanillic acid (≥97%), *α*-pinene (≥98%), *β*-myrcene (≥90%), d-limonene (≥97%), *γ*-terpinene (≥95%), *α*-terpineol (≥95%), *β*-famesene (≥99%), linalool (≥97%), *α*-farnesene (≥99%), nerol (≥97%), hexanal (≥98%), and 3-octanol (≥98.5%), were purchased from Sigma–Aldrich (Auckland, New Zealand). Hesperidin (HPLC grade) and *p*-hydroxybenzoic acid (HPLC grade, ≥99%) were purchased from Abcam Inc. (Melbourne, VIC, Australia).

### 2.2. Preparation of Mandarin Juice and Feed Solution for Spray Drying

All of the mandarin fruits were immediately cleaned with tap water upon arrival at the lab. Each mandarin cultivar was prepared into three sample types, i.e., fruit with removed peel (A1 and RS1), fruit with removed flavedo (A2 and RS2), and whole fruit (A3 and RS3), as shown in Figure S2. All of the samples from both cultivars were processed into juices while using a slow juicer (HUROM, South Korea). The obtained juices were kept in an airtight food container and then stored in a freezer at −20 °C prior to experiments. 

Before the preparation of feed solution for spray drying, several steps were conducted to overcome the gelling behaviour of pectin in mandarin peel. Firstly, the juice samples were blended with Milli-Q water (1:1.5, *w*/*w*) while using a Vitamix blender (Cleveland, OH, USA), followed by sonication with an ultrasonic homogenizer (OMNI Sonic Ruptor, OMNI International, Kennesaw, GA, USA) at a power of 70 W and a pulse rate of 70% for 2 min. Subsequently, the obtained juice was vacuum filtered while using a Büchner funnel that was covered with a filter paper (5–8 µm pore size, Micro Science, Auckland, New Zealand). 

According to the preliminary experiments, a mixture of MD and GA at the ratio of 1:1 (*w*/*w*) was selected as the carrier material for the spray drying of the juice samples. The carrier matrices were mixed with the mandarin juice samples to achieve a core/wall ratio of 1:2 (*w*/*w*). The mixture was stirred at 800 rpm for 15 min. while using a magnetic stirrer, and then stored at 4 °C before spray drying. 

### 2.3. Spray Drying of Juice Samples

Spray drying was performed using a MFJ spray dryer (Dong-Concept New Material Technology Co., Ltd., Nantong, China), as presented in Figure 1. The feed solution was placed into a stainless steel reservoir and then atomized into the spray dryer by a microfluidic nozzle (Φ 75 µm), as described in our previous studies [15,21]. The drying conditions were optimized, as follows: cooling air flow rate at 250 L/min., inlet air temperature of 200 °C, outlet air temperature of 88 °C, nozzle driver frequency being 10 kHz, and the compressed air pressure being set as 0.5 kg/cm^2^. The spray-dried mandarin juice powders, after collection, were transferred into airtight tubes that were subsequently flushed with nitrogen and then sealed by parafilm. All of the samples were stored in a desiccator and then kept at 4 °C until further analysis.

### 2.4. Properties of the Mandarin Juice Powders

#### 2.4.1. Water Activity

The water activity (a_w_) of the powders was measured while using a water activity meter (TH-500; Novasina, Lachen, Switzerland). 

#### 2.4.2. Bulk and Trapped Density

Bulk density (*ρ*_bulk_) and trapped density (*ρ*_tap_) were measured according to a previous study with slight modification [22]. Briefly, the spray-dried mandarin juice powders were weighted and carefully placed into a graduated 10 mL cylinder without touching the interior wall. The volume that was occupied by the powder was recorded as V_o_. The same cylinder filled with powder was then manually tapped 100 times, and the new occupied volume was recorded as V_f_. The *ρ*_bulk_ and *ρ*_tap_ were calculated while using Equations (1) and (2), respectively.

(1) $$\rho_{\mathrm{bulk}}\;\left(g/\mathrm{mL}\right)\;=\frac{powder\;mass}{V_{o}}$$

(2) $$\rho_{\mathrm{tap}}\;\left(g/\mathrm{mL}\right)\;=\frac{powder\;mass}{V_{f}}$$

The Hausner’s ratio and Carr index were calculated according to Equations (3) and (4), respectively [23].

(3) $$Hausner’s\;ratio=\frac{\rho \mathrm{tap}}{\rho \mathrm{bulk}}$$

(4) $$Carr^{\prime }s\;index\;\left(\%\right)=\frac{\rho \mathrm{tap}-\rho \mathrm{bulk}}{\rho \mathrm{tap}}\times 100\%$$

#### 2.4.3. Solubility and Hygroscopicity

The solubility of the powder samples was conducted according to Rigon & Zapata Noreña [24], and it was calculated while using the following Equation (5).

(5) $$Solubility\;\left(\%\right)=\frac{M_{a}-M_{b}}{0.25}\times 100$$

where $M_{a}$ is the mass of the beaker plus sample after drying at 105 °C; and, $M_{b}$ is the initial mass of the beaker

The hygroscopicity of powder samples was conducted according to literature with slight modification [25]. Briefly, 1 g of spray-dried sample was accurately weighted into an aluminium cap. The samples were then kept in a desiccator with consistent humidity (RH 75%, achieved by saturated sodium chloride solution) at room temperature (25 ± 2 °C), until a constant weight was obtained for each sample. The hygroscopicity was expressed as gram of adsorbed moisture per 100 g of powder, as below:

(6) $$Hygroscopicity\;\left(g/100g\right)=\left(\frac{m_{2}-m_{1}-m_{0}}{m_{0}}\right)\times 100$$

where $m_{2}$ is the constant weight obtained after storage; $m_{1}$ is the weight of the aluminum cap; and, $m_{0}$ is the initial sample weight.

#### 2.4.4. Colour Measurement

The colour of the powder samples was measured while using a colorimeter (CR-300, Konica Minolta, Tokyo, Japan). Three indices (L*, a*, and b*) was recorded after each measurement, where L* denotes the lightness (0, black; 100, white), b* indicates the variation from blue (−60) to yellow (60), and a* represents the variation from green (−60) to red (60). The colour intensity in terms of the chroma and hue angle was further acquired from the value of L*, a*, and b* [23].

### 2.5. Morphological Observation by Scanning Electron Microscopy (SEM)

The microstructure of the powder samples was observed while using a SEM (TM3030Plus, Hitachi Ltd., Tokyo, Japan) following the method from our previous studies [16,21]. The powders were attached to an aluminium stub while using a double-side adhesive tape and further sputter-coated with a thin layer of gold palladium. The overall appearance, surface, and cross section of the powders were observed by SEM operated at 5 kV, coupled with 180–1500× magnification. The images that were acquired from SEM were further processed while using Image J software (US National Institutes of Health, Bethesda, MD, USA). 

### 2.6. Determination of Phenolic Compounds by High Performance Liquid Chromatography (HPLC)

Before analysis, each powder sample was reconstituted in Milli-Q water to achieve a similar total solid content as the respective juice sample [15]. The identification and quantification of phenolic compounds were conducted using a HPLC system (HP Agilent 1100 series, Agilent Technologies, Santa Clara, CA, USA) that was equipped with a diode array detector (DAD). A C_12_ column (Synergi Max-RP 80 Å, 4 µm particle size, 250 × 4.6 mm, Phenomenex, Torrance, CA, USA) was applied for phenolic separation. Two mobile phases, A, 0.1% formic acid in Milli-Q water (*v*/*v*), and B, 0.1% formic acid in acetonitrile (*v*/*v*), were used at a constant flow rate of 1 mL/min. The mobile phases were programmed, as follows: 0 min., 5% B (95% A); 10 min., 15% B (85% A); 20 min., 25% B (75% A); 30 min., 35% B (65% A); 32 min., 50% B (50% A); 36 min., 25% B (75% A); 38 min., 5% B (95% A); and, 40 min., 5% B (95% A). The injection volume was 20 µL, the column was maintained at 25 °C, and the detector wavelength was set as 280 nm.

A stock solution for phenolic standards was prepared by dissolving chlorogenic acid (25 mg), p-hydroxybenzoic acid (25 mg), vanillic acid (25 mg), sinapic acid (25 mg), and hesperidin (25 mg) into 25 mL of methanol. The stock solution was then diluted into different concentrations in order to construct standard curves for the quantification of phenolic compounds the samples. The limit of detection (LOD) and limit of quantification (LOQ) of each standard were calculated, as reported by Chen, Buchanan, and Quek (2019) [26]. The precision of the method was evaluated by determining the repeatability (intra-day) and reproducibility (inter-day), and the results were reported as relative standard deviation (RSD%). The intra-day precision was performed by comparing the analysis that was conducted on the same day, whereas the inter-day precision was conducted on three continuous days [26]. 

### 2.7. Characterization of Aroma Compounds by Headspace Solid-Phase Micro-Extraction and Gas Chromatography-Mass Spectrometry (HS-SPME-GC-MS)

The reconstituted samples and original juices (2 mL) were transferred to a 20 mL headspace vial that was sealed with a screw cap. Ten microliter of isotopically labelled standard mixture containing *n*-hexyl-2,2,3,3,4,4,5,5,6,6,6-d_11_-alcohol (0.40 mg/mL), hexanal-d_12_ (0.022 mg/mL), ethyl hexanoate-d_11_ (0.39 mg/mL), hexanoic-d_11_ acid (2.75 mg/mL), α-terpineol-d_3_ (0.025 mg/mL), (±)-linalool-d_3_ (0.025 mg/mL), and 2-phenyl-d_5_-ethanol (2.9 mg/mL) was supplemented into each sample as internal standards (IS). The sealed vial was incubated for 15 min. at 40 °C in a shaking heated cube, and the released volatiles were then extracted by a SPME fibre (2 cm, 24-Gauge, 50/30 µm, Supleco, Bellefonte, PA, USA) that was coated with divinylbenzene/carboxen/polydimethylsiloxane (DVB/CAR/PDMS) at the same temperature for 45 min. After extraction, the fibre was desorbed in the GC injector for 5 min. at 250 °C. 

The volatile compounds were analysed while using a GC-MS system (Shimadzu GCMS-QP2010 Plus, Kyoto, Japan) that was equipped with an Agilent DB-WAX capillary column (30 m × 0.25 mm i.d. × 0.25 μm df) (Santa Clara, CA, USA), as reported in our previous studies [27,28]. The conditions of the column were set, as follows: the oven temperature started from 40 °C for 5 min., increased to 80 °C at 5 °C/min. and held for 5 min., raised to 160 °C for 5 min. (5 °C/min), and, finally, ramped to 230 °C at 10 °C/min. for another 2 min. The mass spectrometer was performed in electron ionization mode at 70 eV with a full scan from 35 to 350 *m*/*z*.

The volatile compounds were identified through the comparison of the obtained mass spectra and retention indices (RIs) with those that were recorded in the NIST 14 library and NIST Chemistry webbook (https://webbook.nist.gov/chemistry/cas-ser/). Key volatile compounds were further confirmed with the available authentic standards. The RIs for the identified compounds and standards were calculated according to a previous study while using the C_7_-C_30_ mixed saturated alkanes as reference [29].

For quantification, a synthetic matrix was made in 100 mL of Milli-Q water containing 4.2 g of sucrose and the pH was further adjusted to 3.5 (pH of original mandarin juice). A stock solution containing *α*-pinene (663.6 µg/mL), *β*-myrcene (320.4 µg/mL), d-limonene (1131 µg/mL), *γ*-terpinene (1320 µg/mL), *α*-terpineol (47.1 µg/mL), *β*-famesene (698.4 µg/mL), linalool (616.4 µg/mL), nerol (32.8 µg/mL), hexanal (63.5 µg/mL), and 3-octanol (79.4 µg/mL) was prepared in the synthetic matrix, which was further diluted into different concentrations for quantification purpose. All of the standard solutions were added with the same amount of IS and then analysed under the same condition as for mandarin samples. The quantification of volatile compounds was conducted while using calibration curves, as shown in Table S1, where the *x* axis denotes the ratio of concentration between the analyte and the IS, and the *y* axis represents the ratio of peak area between the analyte and IS (A_x_/A_is_, analyte peak area/IS peak area). For compounds without available corresponding authentic standards, they were quantitated using the calibration curves of the standards with the same functional groups and/or numbers of carbon atoms. 

### 2.8. Statistical Analysis

Statistical analysis was achieved using SPSS Statistics 25 (IMB, Armonk, NY, USA). The data were compared with Duncan’s multiple range test while using one-way analysis of variance (ANOVA) and they were considered to have a significant difference at *p*-value of >0.05. The software of Unscrambler (version X 10.4, CAMO ASA, Oslo, Norway) was used to perform principal component analysis (PCA).

## 3. Results and Discussion

### 3.1. Physical Properties of Mandarin Juice Powders

#### 3.1.1. Colour Differences among Powder Samples

The colour of the spray-dried powder is important, as it determines the quality and sensory attractiveness of the products [19]. Table 1 shows the colour attributes for the spray-dried mandarin juice powders prepared from the Afourer and Richard Special mandarin fruits (A1, A2, A3, RS1, RS2, and RS3, as described in Section 2.2). Generally, the L* value (lightness) for all of the samples ranged from 85.11 to 88.30, the a* value (redness) varied from 3.39 to 9.26, and the b* value (yellowness) ranged from 30.40 to 54.68 (Table 1). The results indicated that the mandarin juice powder samples showed higher differences in redness (a* value) and yellowness (b* value) than lightness (L* value).

Furthermore, the chroma and the hue angle (representing the colour saturation and the colour perception, respectively) were calculated in order to better understand the colour differences among all of the samples. Powders that were made from the whole fruit samples had the highest colour saturation (chroma value of 52.71 for A3 and 54.28 for RS3), followed by the samples from the flavodo-removed fruits (A2 & RS2; 48.08 & 43.54), and those from the peel-removed fruits showed the least colour saturation (A1 & RS1; 29.54 & 37.95), as shown in Table 1. This suggested that the colour pigments in the mandarin peels have a significant contribution to the colour of the powders. The values of hue angle at 0°, 90°, 180°, and 270° indicate the colour of red, yellow, green, and blue, respectively [23]. According to the results (Table 1), all of the samples were strongly characterized by yellow colour as their hue angle values are close to 90° (73.69° to 86.52°). The samples prepared from the peel-removed fruits (A1 & RS1) had the lightest yellowness (A1, 73.94°; RS1, 73.69°), while the powders that were derived from the whole mandarin fruits (A3 & RS3, 86.50° & 84.69°) showed the most intensive yellow colour (*p* < 0.05).

The literature reported that inlet air temperature could affect the colour of the spray-dried powders due to the non-enzymatic browning reaction during processing [19]. Besides, the type of carrier or wall material added in the feed solution, together with their concentrations, could have an impact on the colour of the powder produced [14]. In this study, we have used the same wall material and spray drying conditions for all of the samples; therefore, the colour difference across the mandarin juice powders could be mainly attributed to the sample types (that were prepared with/without mandarin albedo and/or flavedo). The bright orange-yellow colour of mandarin is related with the levels of carotenoids, which normally present at a higher concentration in peel than in pulp (300 μg/g FW vs. 40 μg/g FW) [1]. Therefore, the removal of peel or flavedo during sample preparation could greatly reduce the yellowness in the mandarin juice powders.

#### 3.1.2. Water Activity of the Powders

Water activity (a_w_) reflects the available free water in food system, which is responsible for microbial growth and biochemical reactions [30]. The food system with the a_w_ value below 0.6 is regarded as safe from microbial deterioration [19], and a_w_ values that are below 0.25 represent minimized undesirable changes, including non-enzymatic browning reactions, lipid oxidation, enzymatic activity, and hydrolytic reactions in foods during storage [31]. Our results show that the a_w_ values of the mandarin juice powders were in a range of 0.14 to 0.25 (Table 2). These values are significantly lower than 0.6, the level that is required for microbial growth. This indicates that all of the spry dried powder samples produced were microbiologically and chemically stable.

#### 3.1.3. Density of the Powders 

Bulk and trapped density are crucial parameters in powder production and they have been used to estimate the cost involved in powder packaging and transportation [17]. In the current study, the bulk and trapped densities obtained for all the juice powders were between 0.5 to 0.6 g/mL and 0.6 to 0.7 g/mL, respectively (Table 2). These results were similar to those that were reported in our previous study on Noni juice, while using the same MFJ spray dryer [16]. The bulk density of the A2, A3, RS2, and RS3 powder samples were higher in comparison to that of the A1 and RS1 powders. This phenomenon could be attributed to the differences in the pectin content of the juice samples before spray drying. In mandarin fruit, the flavedo contains the highest concentration of water-soluble pectin, followed by the albedo and juice vesicles [32]. Additionally, the average molecular weight of pectin in each part of mandarin could be varied significantly. Pectin in the albedo and flavedo have higher molecular weight, i.e., 353 kDa and 295 kDa, respectively. In contrast, pectin in the juice vesicle shows a lower value of 189 kDa [32]. Therefore, powders that were prepared from the whole mandarin fruit juice (A3 and RS3) and those from the juice samples with albedo only (A2 and RS2) contained a higher concentration of pectin that have greater molecular weights. This could explain the higher density of those samples when compared to the A1 and RS1 samples, where both albedo and flavedo were removed.

#### 3.1.4. Flowability and Cohesiveness of Powders

The Carr’s index is related to the powder flowability, while the Hausner’s ratio indicates the cohesiveness of the spray-dried powders. From Table 2, the Carr’s index showed a relatively bigger range among all of the spray-dried powders (from 17.14 to 22.05%), while the Hausner’s ratio for all samples only varied slightly from each other (from1.21 to 1.28). No significant differences were observed for these two indices for the samples produced from the flavedo-removed juices (i.e., A2 vs. A3) and the whole fruit juices (i.e., RS2 vs. RS3). The flowability of microcapsules is highly related to the particle size. Smaller particle size represents s higher surface area to mass ratio, and it is often responsible for good flowability in powder samples [33]. The flowability and cohesiveness of powders could also be influenced by the moisture content of the samples. A higher moisture content could be associated with a greater force between particles, due to the formation of liquid bridge, thus contributing to a poorer flowability [23]. 

#### 3.1.5. Solubility of Powders

The solubility of the spray-dried powders was around 74% with no significant difference across all the samples (*p* > 0.05; Table 2). This agrees with the previous study on spray-dried watermelon powders [20]. 

#### 3.1.6. Hygroscopicity of Powders

Hygroscopicity reflects the ability of powders to absorb moisture from the ambient environment. It has been commonly treated as another important parameter in order to determine the powder flowability [34]. A higher hygroscopicity value basically indicates a stronger tendency for the spray-dried powders to form a caking and stickiness status during shelf-life storage. The current results showed that the hygroscopicity ranged between 17.45 and 18.53 g/100 g (Table 2) for different juice powders. The values were similar to those that were reported on spray-dried blackberry [24], and sour cherry juice [25], but slightly higher than the values that were reported for pineapple and carambola powders [20]. 

### 3.2. SEM Imaging Analysis

**Figure 2:**
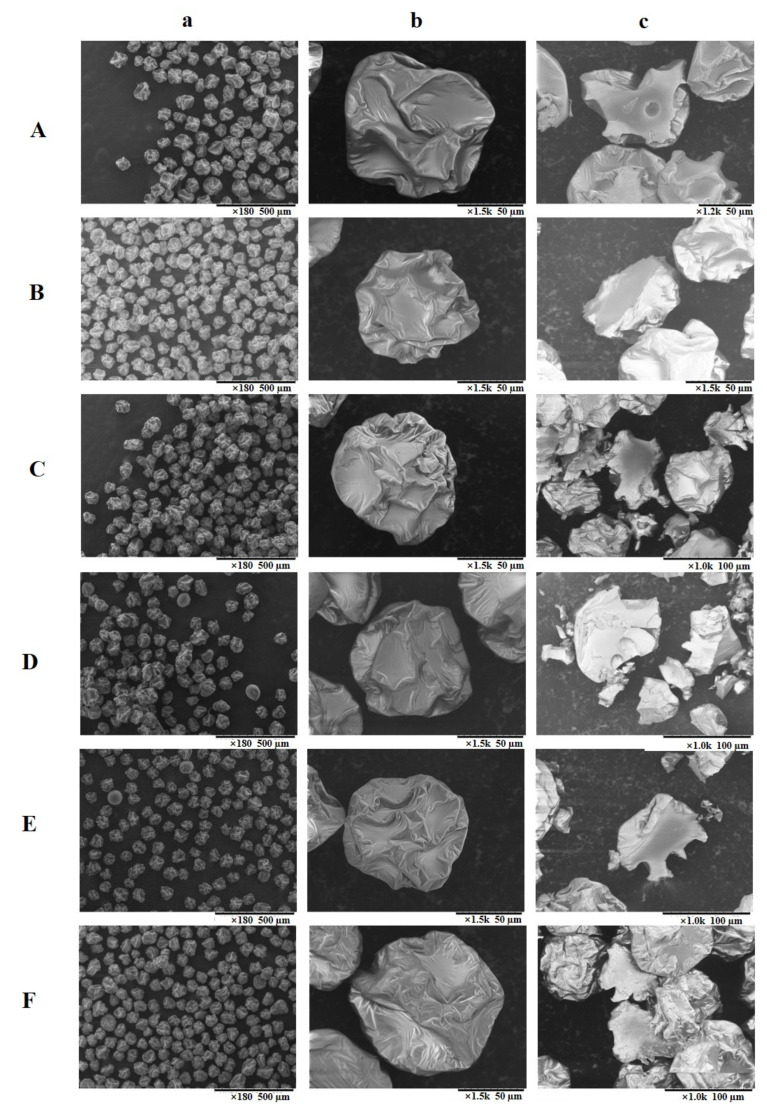
Scanning Electron Microscopy (SEM) micrograph of Afourer mandarin and Richard Special mandarin microcapsules. (**a**), 180× magnification; (**b**), 1500× magnification; (**c**), 1000× magnification; (**A**), A1 microcapsules; (**B**), A2 microcapsules; (**C**), A3 microcapsules; (**D**), RS1 microcapsules; (**E**), RS2 microcapsules; and, (**F**), RS3 microcapsules.

Figure 2 shows the microstructure of the spray-dried powders, as observed using SEM. The images in the first column (a) showed an overview of the spray-dried powders, and those in column (b) and (c) provided a detailed observation on a single particle and its cross section, respectively.

The mean particle diameters of sample A1, A2, and A3 were calculated as 100.27 ± 10.05, 84.13 ± 8.42, and 78.04 ± 9.67 µm, respectively. For powders of RS1, RS2, and RS3, the particle mean diameter was 99.57 ± 8.76, 81.15 ± 8.34, and 71.37 ± 6.52 µm, respectively. Overall, a smaller particle size was observed for the mandarin powder that was made from the whole fruit juice. This could be associated with the presence of pectin in the mandarin peel, which could participate as the wall material during spray drying. The literature [35] showed that the addition of pectin in the wall materials resulted in producing spray-dried powders with a much lower mean diameter as compared to the use of maltodextrin as a wall material (3.2–5.5 μm vs. 22.9–45.9 μm), which could be responsible for the phenomenon that was observed in the current study. 

The spray dried particles that were produced in this study exhibited an intact surface without fissures (Figure 2, column a). This indicates that the wall material has provided an effective protection against the possible oxidation and degradation of bioactive compounds in the mandarin juices. The morphology of the mandarin juice powders was discrete, and it showed uniformity in particle size and shape, which was very different from the uneven and agglomerated morphology of spray-dried powders that were found in most of the previous studies [14,22,23]. The similar surface morphology of each microcapsule could ensure a uniform quality of the final product, and such desirable powder morphology was due to the advantages of the special nozzle that was designed for the MFJ spray dryer [18]. 

Particles from the whole fruits (column b, C and F) exhibited a relatively severer winkled surface with deeper indentations and bigger bulges, while spray-dried powders that were prepared from the peel-removed mandarin juices showed relative mild indentations (Figure 2, column b, A and D). Zhang et al. observed a similar morphology feature when studying Noni juice particles produced by MFJ spray drying, and they ascribed this phenomenon to the properties and different concentrations of wall materials that are used during spray drying [21]. However, in the current study, the morphology difference could not be related to the effect of wall materials or drying conditions, as these parameters were exactly the same for all of the samples. In this regard, a possible reason accounted for the morphology difference in these samples could be due to the variation of sugar and organic acid contents in different mandarin juices. The presence of sugars and acids could lessen the surface tension of particles during spray drying and, thus, yielding shallower and less rugged surface troughs [36].

Generally, the interior surface was smoother than the exterior surface (column c and b). A solid inner was observed for all spray-dried particles, and some small vacuoles were present in the interior of the A1 and RS1 particles (A and D). The literature reported that two drying patterns could exist during spray drying of a particle, i.e., “dry shell” route and “wet shell” route [17]. These two routes largely decide the interior morphology of the final particles, determining whether a solid or a hollow inner structure would be formed. After the formation of the crust, which is the second stage of the drying process from droplet-to-particle [21], powders will follow one of the routes, depending on the nature of the crust formed. The “dry shell” route leads to the formation of solid particle when appropriate drying temperature is applied, while the “wet shell” gives hollow particles that are susceptible to inflation when higher inlet temperature is applied. Based on these explanations, the mandarin juice powders presented in the current study mainly underwent the “dry shell” route. Besides, the small vacuoles could be related to the foaming property of protein that was present in the wall material of GA at a concentration of approximately 2% [36].

### 3.3. Retention of Phenolic Compounds during Spray Drying

A HPLC system equipped with a DAD was employed to analyse the major phenolics in the mandarin juices and the spray-dried powders from both Afourer and Richard Special cultivars in order to investigate the effect of sample types on the retention of individual phenolic compound. The method validation parameters, including the repeatability, intermediate precision, regression coefficient, LOD, and LOQ, are shown in Table S2. 

Table 3 shows the concentrations of major phenolics in the juice and powder samples. It has been widely reported that mandarin fruits have an abundant flavanone profile that is dominated by hesperidin [37], and this was confirmed by the results that were obtained for the Richard Special cultivar in the current study (15 to 180.5µg/100 mg DW). Sinapic acid was present as the predominant phenolic compound with the concentration ranging from 406.9 to 913 µg/mg DW in both mandarin juice samples and powders of the Afourer cultivar. Some other flavonoids, such as naringin, rutin, and eriocitrin, which have been identified as major flavonoids in mandarin fruits by previous researchers, were not detected in our samples [3]. This could probably be explained by the diversity of mandarin varieties, as the literature indicated that the phenolic profile could be genetically controlled [37]. Additionally, the effect of environmental factors, including climate, soil properties, humidity, and light, could also be responsible for the diverse phenolic profile for mandarins [3].

With regards to the retention of phenolic compounds, sinapic acid decreased significantly (*p* < 0.05) in all samples, except RS3, after drying. This decline could be attributed to the degradation of phenolic compounds. The hesperidin had an opposite trend, showing a dramatic increase in the spray-dried samples, especially for the RS1 sample, giving a retention value of 594.6%. The results showed that thermal processing did not affect the stability of hesperidin, in agreement with a previous study that was conducted by Dhuique-Mayer et al. [38]. They reported no significant loss of hesperidin under heat treatment at 90 °C for 240 min. The increase of phenolic acids, for example, *p*-hydroxybenzoic acid for RS1 (retention of 225%), coumaric acid for all Richard Special samples (retention of 254–287%), and vanillic acid for all samples, except RS1 (retention of 101–199%), was associated with the release of phenolic aglycones from their glycosidically bound precursors, due to the disruption of cell membranes under thermal treatment [39]. This was supported by the findings of a previous study [37], that the concentrations of the bound-form phenolic acids in citrus *reticulata Blanco* were considerably higher than their free counterparts, especially for chlorogenic acids, which has a bound form concentration that is 5.6 times higher than that in the free fraction (264.93 µg/g DW vs. 46.68 µg/g DW). 

When comparing the total five phenolics concentrations in all samples, the spray-dried whole fruit powders (A3 and RS3) showed the highest value (1023 µg/100 mg DW, and 314 µg/100 mg DW, respectively), followed by the powders that were prepared from the flavedo-removed mandarins (A2 and RS2, 808.6 and 179 µg/100 mg DW, respectively). The powders that are prepared from the peel-removed fruit juice (A1 and RS1, 653 and 146 µg/100 mg DW, respectively) have the lowest total phenolic contents. These findings reaffirmed that the preparation of spray-dried powders from whole mandarin fruit juices has an advantage, as they retain higher phenolic contents. 

### 3.4. Volatile Compounds in Mandarin Juices and Juice Powders 

Volatile compounds play a critical role in fruit flavour and, thus, have been considered to be an important contributor to the sensory quality of fruit products. However, due to thermal treatment, key aroma compounds, such as esters, terpenes, aldehydes, and alcohols, could easily escape from the original fruit juices during spray drying. 

In this study, a total of 26 aroma compounds were quantified in all of the mandarin samples (Table 4). These compounds were grouped into three categories, namely terpenoids, aldehydes, and alcohols. Terpenoids are important compounds for plants to defend against pathogens, insects, and competitors, as well as to attract seed disseminators and pollinators [40]. They have also been reported as major volatile compounds in mandarin juice as well as the essential oils that were extracted from mandarin peels [41]. The current study revealed a similar finding that terpenoids were the most abundant group, comprised of 21 compounds in both the original juices and reconstituted juice samples with the concentrations ranging from 12,355 µg/L to 864,558 µg/L. Among the 21 terpenoids that were detected in this study, five of them, namely *α*-cubebene, *cis*-*β*-elemene, *β-*famesene, *α-*famesene, and nerolidol, belong to sesquiterpenes, while the rest are assigned to monoterpenes.

d-limonene was discovered with the highest concentration (11,128 to 672,178 µg/L), accounting for 70–96% of the total terpene concentration. It has also been reported as one of the most important aroma-active compounds in citrus fruits, being responsible for a pleasant citrus-like odour [42]. In addition, d-limonene is listed as “Generally Recognized as Safe (GRAS)” in the Federal Regulations Codes and, therefore, has been widely used as a flavoring agent for food items of soft drinks, puddings, ice creams, fruit juices, and chewing gums, etc., as well as a fragrance additive in personal hygiene productions (soaps, perfumes, etc.) [43]. It is noted that there is no significant change in the concentration of d-limonene between the original mandarin juice and spray-dried powder (Table 4), which indicated a generally good preserving effect of the wall materials on juice flavour. In addition, *β*-myrcene, *γ*-terpinene, *α*-pinene, and *β*-pinene are also major flavour compounds in mandarin samples, which was accordance with the free volatile profile of mandarin juice that was reported by Ren et al. [41]. However, the retention of these compounds showed different trends across samples. For example, the concentration of *α*-pinene in RS2 powder after spray-drying significantly increased from its original juice sample (juice vs. powder, 688 µg/L vs. 6613 µg/L), while, in the spray-dried RS1 sample, it dropped to the half value observed in the original juice (juice vs. powder, 218.4 µg/L vs. 112.2 µg/L). *β*-Myrcene, which was characterized by the sweet and balsamic odour, showed a decrease in concentration among all of the spray dried samples, except for RS1 and RS2. 

There are some reasons that are related to these phenomena. Firstly, the high temperature applied for spray drying could increase the relative volatility of volatile compounds during processing and thus, leading to the loss of volatiles in the final products. This was supported by a previous study on the odour retention of noni juice powders that were prepared by MFJ spray dry and freeze dry, respectively [15]. They discovered that the retention of octanoic acid and hexanoic acid in the freeze-dried powders were 90.2 and 90.1%, respectively, while, in the spray-dried microcapsules, their retentions were all below 50%. The difference was ascribed to the increased volatility of aromatic compounds induced by spray-drying [15]. On the other hand, the degradation of volatiles during thermal treatment might also result in decreased concentrations in powders.

Secondly, glycosidically bound volatiles are widely found in most fruits with higher concentrations than their free counterparts [27], and mandarins showed a more abundant profile of glycosidic volatiles than other citrus species, i.e., grape fruit, as reported by Ren et al. [41]. During spray drying, the acidic juice matrix with the aid of thermal processing condition could lead to the liberation of free volatile aglycones from their corresponding glycosidic precursors. Consequently, this will cause the increase of aroma compounds in the final powders. Finally, volatile compounds would go through transformation under heat treatment, for example, linalool and d-limonene would transform into α-terpineol [42], and this could contribute to the accumulation of specific compounds.

*α*-Terpineol and terpinene-4-ol are reported as two off-flavour compounds in mandarin juices [42]. In this study, the heat that is induced by spray-drying did not cause their augmentation. Especially for *α*-terpineol, its concentration in powder samples were much lower (*p* < 0.05) than that in the initial mandarin juices. This indicated that microencapsulation might have a masking effect on the unpleasant odour that is present in mandarin juices.

In addition, three aldehydes (hexanal, nonanal, and decanal) and two alcohols (1-heptanol and 1-octanol) were found in both juices and microcapsules samples. Their total concentrations were considerably high in the samples from the whole mandarin fruits, i.e., A3 (5138 µg/L for juice and 3811 µg/L for powders) and RS3 (5452 µg/L for juice and 9735 µg/L for powders), followed by the samples from the flavedo-removed mandarins, i.e., A2 (943.5 µg/L for juice and 772 µg/L for powders) and RS2 (1026 µg/L for juice and 1214 µg/L for powders). This suggested that the peel of mandarin was also high in aldehyde and alcohol compounds.

By comparing the two cultivars, no significant difference was observed for the total concentrations of terpenoids and aldehydes between the A1 & RS1 and the A3 & RS3 juice samples (Table 4). For the juices of A2 and RS2, the Afourer cultivar showed a significantly higher concentration in total terpenoids (134,467 µg/L vs. 35,916 µg/L), but lower content of alcohols (68.2 µg/L vs. 128.5 µg/L) than the Richard Special cultivar. In the juice powders, the Richard Special cultivar had significantly higher contents of terpenoids, aldehydes and alcohols in flavedo-removed and whole fruit samples (*p* < 0.05) (Table 4).

Overall, the highest concentration of aroma compounds was found in the whole mandarin fruit and the lowest in the pulp juice for both of the cultivars. After spray-drying, the concentration of volatile compounds with pleasant odour (d-limonene, *β*-myrcene, and *α*-pinene) were well retained, whereas, for the off-odour compounds (α-terpineol and terpinene-4-ol), their unpleasant notes were masked to some extent by the microencapsulation.

### 3.5. Distribution of Phenolic and Aromatic Compounds in Different Samples

**Figure 3:**
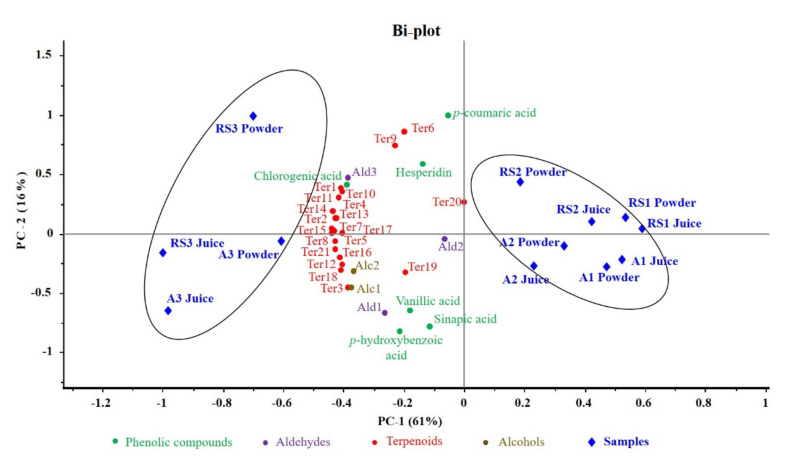
Principle component analysis (PCA) of phenolic and aromatic compounds in Afourer and Richard Special mandarin juices and spray-dried powders.

A PCA map presented the distribution of phenolic and aroma compounds among mandarin juices and spray-dried powders that were prepared from the two cultivars studied (Figure 3). The PCA results were plotted in two main principal compounds (PCs) and they explained 77% of the total variance. The PC-1 and PC-2 accounted for 61% and 16%, respectively. All of the volatile variables were color-coded according to chemical classes, and the codes are indicated in Table 4.

PC-1 clearly separated all 12 samples into two groups. Samples that were produced with whole mandarin fruits, both the initial juice samples and spray-dried powders (type 3 samples, including A3 juice, RS3 juice, A3 powder, and RS3 powder) were located in the negative side of PC-1. Samples that were prepared from the peel-removed mandarins (type 1 samples consisting of A1 juice, RS1 juice, A1 powder. and RS1 powder) and the flavedo-removed fruits (type 2 samples comprised of A2 juice, RS2 juice, A2 powder, and RS2 powder) were distributed in the right side of PC-1. In addition, type 1 of type 2 samples can also be differentiated. All of the flavedo- removed samples (type 2), regardless of juices or powders, were closer to the middle of PCA map, while the type 1 samples were registered in the more positive side of PC-1 axis (Figure 3). Furthermore, PC-2 generally separated out the mandarin cultivars, as all of the Richard Special samples (except RS3 juice) were plotted in the upper side of the PCA map, while the Afourer juices and powders all appeared at the lower part.

Phenolic and volatile compounds that were reported in the present study were clustered in the left side of PCA plot, closer to the RS3 and A3 samples. This indicated these phytochemicals were highly correlated with the whole mandarin fruits, which was in accordance with the high concentrations of phenolics and volatiles in the juices and spray-dried microcapsules from the whole fruit (Table 3 and Table 4). To be specific, most of the terpenoids, e.g., *β*-pinene, linalool, *α*-farnesene, geranyl acetate, *β*-myrcene, and geraniol, showed higher correlations with the RS3 and A3 juices/powders than the aromatic aldehydes (hexanal, nonanal, and decanal) and alcohols (1-heptanol and 1-octanol), as well as all of the phenolic compounds concerned in the current study. Overall, the PCA map clearly revealed that different mandarin cultivars and various sample types would have pronounced impacts on the volatile and phenolic profile of spray-dried microcapsules, and the microcapsules made from whole fruit juice have superiority in terms of the phytochemical abundance.

## 4. Conclusions

In the current study, we systematically compared the physical properties, phenolic retention, and volatile profile of three types of mandarin powders that are derived from the whole fruit juices (A3 & RS3), the flavedo-removed fruit juices (A2 & RS2), and the peel-removed fruit juices (A1 & RS1). Overall, the A3 and RS3 powders had a higher yellow colour than the other powders (A2 & RS2 and A1 & RS1). For density, solubility, and water activity, no significant differences were observed between the mandarin powders that were prepared from the whole fruit juices (A3 and RS3) and the flavedo-removed fruit juices (A2 & RS2). Regarding the microstructure of powders, the whole fruit juice powder exhibited a relatively severer winkled surface with deeper indentations and bigger bulges. Furthermore, the wall materials showed a satisfactory protection effect on individual phenolic compound, and a significantly higher amount of total phenolic compounds was observed from the powders that were produced from whole fruit juices (A3, 1023 µg/100 mg vs. A2, 809 µg/100 mg vs. A1, 653 µg/100 mg; RS3, 314 µg/100 mg vs. RS2, 179 µg/100 mg vs. RS1, and 146 µg/100 mg). After spray drying, volatile compounds with pleasant odours (d-limonene, *β*-myrcene, and *α*-pinene) were well retained in the particles, whereas the unpleasant notes from off-odour compounds (α-terpineol and terpinene-4-ol) were sufficiently masked. The whole fruit powders had a significantly higher content of total volatile compounds (A3, 775,558 µg/L vs. A2, 125,617 µg/L vs. A1, 12,590 µg/L; RS3, 865,023 µg/L vs. RS2, 286, 763 µg/L vs. RS1, 22,902 µg/L). This research provided an environment-sustainable method for efficiently utilising bioactive compounds from whole mandarin fruits, meanwhile minimising the by-products generation during mandarin fruit processing.

## Figures and Tables

**Table 1:** Color attributes of mandarin powders derived from Afourer and Richard Special cultivars.

	L*	a*	b*	Chroma	Hue Angle
**A1 powder**	88.30 ± 0.56 ^a^	8.04 ± 0.65 ^b^	30.40 ± 0.59 ^f^	29.54 ± 2.53 ^e^	74.94 ± 2.71 ^e^
**A2 powder**	86.04 ± 0.29 ^cd^	5.69 ± 0.45 ^d^	50.26 ± 0.40 ^c^	48.08 ± 2.77 ^b^	83.28 ± 0.81 ^c^
**A3 powder**	87.10 ± 0.39 ^b^	3.39 ± 0.34 ^e^	52.57 ± 0.70 ^b^	52.71 ± 0.79 ^a^	86.50 ± 0.28 ^a^
**RS1 powder**	85.11 ± 1.43 ^d^	9.26 ± 0.10 ^a^	37.09 ±0.37 ^e^	37.95 ±1.32 ^d^	73.69 ±2.53 ^e^
**RS2 powder**	85.22 ± 0.50 ^d^	8.97 ± 0.85 ^a^	42.85 ± 0.80 ^d^	43.54 ± 0.85 ^c^	78.67 ± 0.75 ^d^
**RS3 powder**	86.33 ± 0.73 ^bc^	6.79 ± 0.11 ^c^	54.68 ± 0.24 ^a^	54.28 ± 1.23 ^a^	84.69 ± 1.51 ^bc^

A1—peel-removed mandarin fruit from Afourer cultivar; A2—flavedo-removed mandarin fruit from Afourer cultivar; A3—whole Afourer mandarin fruit; RS1—peel-removed mandarin fruit from Richard Special cultivar; RS2—flavedo-removed mandarin fruit from Richard Special cultivar; RS3—Whole Richard Special mandarin fruit; L* represents lightness; b* indicates the blue-yellow component; a* indicates the green-red component; Values are expressed as mean ± standard deviation; Different lower-case letters (a–f) indicate statistical significance (*p* < 0.05) among samples in the same column as analysed by Duncan’s test.

**Table 2:** Properties of spray-dried mandarin juice powders.

	A1 Powder	A2 Powder	A3 Powder	RS1 Powder	RS2 Powder	RS3 Powder
**Water Activity (Aw)**	0.25 ± 0.00 ^a^	0.17 ± 0.01 ^b^	0.20 ± 0.01 ^a^	0.17 ± 0.01 ^b^	0.20 ± 0.003 ^a^	0.14 ± 0.01 ^c^
**Bulk density, *ρ*_bulk_ (g/mL)**	0.56 ± 0.01 ^c^	0.61 ± 0.01 ^a^	0.60 ± 0.01 ^a^	0.57 ± 0.01 ^c^	0.58 ± 0.01 ^bc^	0.60 ± 0.01 ^ab^
**Tapped density, *ρ*_tap_ (g/mL)**	0.71 ± 0.00 ^cd^	0.75 ± 0.02 ^ab^	0.73 ± 0.02 ^bc^	0.69 ± 0.00 ^d^	0.72 ± 0.02 ^c^	0.76 ± 0.02 ^a^
**Carr’s index (%)**	21.48 ± 0.02 ^a^	18.37 ± 0.33 ^bc^	19.00 ± 0.31 ^c^	17.14 ± 0.00 ^c^	21.02 ± 1.41 ^ab^	22.05 ± 1.38 ^a^
**Hausner’s ratio**	1.27 ± 0.02 ^a^	1.23 ± 0.01 ^b^	1.22 ± 0.01 ^b^	1.21 ± 0.00 ^b^	1.27 ± 0.02 ^a^	1.28 ± 0.02 ^a^
**Solubility (%)**	74.19 ± 1.13 ^a^	74.07 ± 1.62 ^a^	73.82 ± 1.02 ^a^	75.30 ± 1.56 ^a^	74.82 ± 1.40 ^a^	73.84 ± 1.28 ^a^
**Hygroscopicity** **(g moisture/100 g solids)**	17.45 ± 0.14 ^c^	17.71 ± 0.21 ^bc^	18.20 ± 0.19 ^ab^	18.02 ± 0.40 ^bc^	17.53 ± 0.48 ^abc^	18.53 ± 0.46 ^a^

A1—peel-removed mandarin fruit from Afourer cultivar; A2—flavedo-removed mandarin fruit from Afourer cultivar; A3—whole Afourer mandarin fruit; RS1—peel-removed mandarin fruit from Richard Special cultivar; RS2—flavedo-removed mandarin fruit from Richard Special cultivar; RS3—Whole Richard Special mandarin fruit; Values are presented as mean ± standard deviation (*n* = 3). Different letters (a–d) in the same row indicate significant statistical difference (*p* < 0.05) as analysed by Duncan’s test.

**Table 3:** Concentration of individual phenolic compound in mandarin juice and spray-dried powders and the retention during spray drying.

	Concentrations (µg/100 mg DW) *^A^*					
	Chlorogenic Acid	Hesperidin	*p*-Coumaric Acid	*p*-Hydroxybenzoic Acid	Sinapic Acid	Vanillic Acid
A1 juice	9.6 ± 0.17 ^b^	44.8 ± 0.72 ^b^	ND ***^C^***	65.9 ± 0.92 ^f^	489.7 ± 7.6 ^c^	44.3 ± 1.1 ^e^
A1 powder	14.1 ± 0.95 ^c^	105.9 ± 0.46 ^h^	ND	69.7 ± 0.96 ^g^	406.9 ± 15.4 ^b^	56.7 ± 2.4 ^g^
***A1 Retention/% ^B^***	***145.8***	***236.4***	***N/A ^D^***	***105.8***	***83.1***	***128.0***
A2 juice	13.68 ± 0.14 ^c^	46.7 ± 0.19 ^b^	ND	70.0 ± 0.46 ^g^	554.1 ± 22.2 ^d^	48.1 ± 0.15 ^f^
A2 powder	17.2 ± 0.88 ^d^	147.6 ± 3.0 ^i^	ND	57.5 ± 2.8 ^e^	529.2 ± 25.2 ^d^	57.1 ± 0.58 ^g^
***A2 Retention/%***	***125.55***	***316.06***	***N/A***	***82.26***	***95.51***	***118.71***
A3 juice	32.8 ± 0.82 ^g^	66.4 ± 1.5 ^e^	ND	91.5 ± 1.5 ^i^	912.6 ± 9.6 ^f^	94.5 ± 1.3 ^h^
A3 powder	28.8 ± 1.2 ^f^	148.5 ± 0.76 ^i^	ND	77.9 ± 4.1 ^h^	664.5 ± 43.9 ^e^	103.6 ± 1.0 ^i^
***A3 Retention/%***	***87.80***	***223.64***	***N/A***	***85.14***	***72.81***	***109.63***
RS1 juice	8.4 ± 0.05 ^a^	15.0 ± 0.27 ^a^	0.80 ± 0.05 ^a^	19.3 ± 0.72 ^bc^	8.7 ± 0.46 ^a^	4.6 ± 0.09 ^a^
RS1 powder	18.9 ± 0.37 ^e^	88.6 ± 0.64 ^f^	2.4 ± 0.03 ^d^	14.7 ± 0.28 ^a^	13.3 ± 1.7 ^a^	8.4 ± 0.53 ^b^
***RS1 Retention/%***	***225.00***	***594.63***	***287.50***	***131.29***	***65.41***	***54.76***
RS2 juice	14.3 ± 0.21 ^c^	61.7 ± 0.46 ^d^	1.06 ± 0.03 ^b^	22.2 ± 0.60 ^c^	17.8 ± 0.17 ^a^	7.4 ± 0.2 ^b^
RS2 powder	28.3 ± 0.42 ^f^	98.9 ± 4.5 ^g^	2.8 ± 0.04 ^e^	18.1 ± 0.98 ^b^	16.2 ± 0.66 ^a^	14.7 ± 0.17 ^c^
***RS2 Retention/%***	***197.90***	***160.45***	***254.55***	***81.53***	***91.01***	***198.65***
RS3 juice	55.8 ± 0.32 ^i^	56.4 ± 0.75 ^c^	1.25 ± 0.04 ^c^	67.9 ± 2.4 ^fg^	28.2 ± 0.29 ^a^	18.5 ± 0.82 ^d^
RS3 powder	51.7 ± 0.38 ^h^	180.5 ± 3.6 ^j^	3.6 ± 0.16 ^f^	29.5 ± 0.51 ^d^	30.2 ± 2.4 ^a^	18.6 ± 0.80 ^d^
***RS3 Retention/%***	***92.65***	***320.04***	***276.92***	***43.45***	***106.74***	***100.54***

A1—peel-removed mandarin fruit from Afourer cultivar; A2—flavedo-removed mandarin fruit from Afourer cultivar; A3—whole Afourer mandarin fruit; RS1—peel-removed mandarin fruit from Richard Special cultivar; RS2—flavedo-removed mandarin fruit from Richard Special cultivar; RS3—Whole Richard Special mandarin fruit; ***^A^*** Values are expressed as mean ± standard deviation; different letters (a–i) in the same column indicate significant statistical difference (*p* < 0.05) between samples; ***^B^*** The retention of individual phenolic compound is calculated according to the average concentration of phenolic compound detected in the spray-dried microcapsules divided by that observed in the corresponding juice sample; ***^C^*** ND, not detected in the sample; ***^D^*** N/A, not applicable.

**Table 4:** Concentration of volatile compounds in mandarin juices and powders.

^A^RI	^B^Compound	^C^Code	Samples	Concentration (µg/L)											
				A1		A2		A3		RS1		RS2		RS3	
				Mean	STD%	Mean	STD%	Mean	STD%	Mean	Std%	Mean	Std%	Mean	Std%
	***Terpenoids***														
1018	*α-Pinene	Ter1	Juice	257.7 ^f^	9.1	4648.1 ^de^	3.4	11,477.3 ^c^	16.5	218.4 ^f^	5.7	688.1 ^f^	10.4	20,751.9 ^a^	8.6
			Powder	118.6 ^f^	9.6	3624.4 ^e^	9.9	15,933.8 ^b^	1.5	112.2 ^f^	9.7	6613.1 ^d^	1.2	23,330.0 ^a^	12.8
1116	*β*-Pinene	Ter2	Juice	122.0 ^f^	9.5	2166.2 ^e^	3.2	15,454.9 ^b^	3.7	N.D.	N/A	350.6 ^f^	6.5	19,847.5 ^a^	9.4
			Powder	^D^N.D.	^E^N/A	1951.6 ^ef^	12.4	14,497.5 ^c^	8.7	9.8 ^f^	9.8	4694.2 ^d^	10.3	13,876.9 ^c^	11.8
1160	*β-Myrcene	Ter3	Juice	721.9 ^de^	2.4	8959.4 ^c^	9.5	36,170.8 ^a^	8.1	87.1 ^e^	8.1	1498.8 ^de^	4.1	31,926.8 ^b^	10.8
			Powder	573.4 ^de^	6.5	1743.2 ^de^	4.2	10,819.7 ^c^	5.9	118.8 ^e^	6.5	2684.5 ^d^	4.0	7685.4 ^c^	10.8
1180	*d-Limonene	Ter4	Juice	11617.3 ^d^	19.4	107,098.5 ^cd^	3.1	587,867.0 ^b^	6.8	12,860.3 ^d^	2.8	27,705.9 ^d^	2.6	699,261.6 ^a^	9.4
			Powder	11128.2 ^d^	1.0	113,068.2 ^cd^	8.3	635,341.3 ^ab^	7.7	21,746.7 ^d^	6.6	264,961.4 ^cd^	10.9	672,178.4 ^a^	0.7
1213	*γ-Terpinene	Ter5	Juice	961.6 ^de^	0.5	6578.5 ^de^	7.4	155,084.6 ^a^	10.6	60.1 ^d^	7.0	3695.6 ^de^	11	67,276.4 ^c^	8.8
			Powder	N.D.	N/A	536.4 ^de^	8.7	73,418.7 ^c^	7.9	N.D.	N/A	122.4 ^d^	13.2	117,129.2 ^b^	11.2
1296	*p*-Mentha-1,5,8-triene	Ter6	Juice	0.8 ^e^	17.2	14.0 ^d^	18.2	25.2 ^cd^	16.9	N.D.	N/A	30.3 ^c^	7.8	24.6 ^cd^	6.9
			Powder	N.D.	N/A	16.7 ^d^	7.4	53.6 ^b^	2.1	N.D.	N/A	29.6 ^cd^	0.9	364.1 ^a^	6.0
1451	*α*-Cubebene	Ter7	Juice	117.5 ^d^	2.4	290.6 ^c^	2.9	672.8 ^b^	8.5	116.0 ^d^	0.1	127.2 ^d^	0.8	812.8 ^a^	9.0
			Powder	115.6 ^d^	2.4	265.5 ^c^	2.6	810.1 ^a^	8.9	119.4 ^d^	6.9	266.7 ^c^	18.2	644.8 ^b^	7.7
1537	*Linalool	Ter8	Juice	65.4 ^f^	14.9	908.7 ^d^	2.5	8106.1 ^a^	2.4	73.4 ^f^	12	781.2 ^de^	2.2	8530.5 ^a^	4.4
			Powder	N.D.	N/A	258.1 ^ef^	4	3319.0 ^c^	6.3	10.5 ^f^	2.2	820.7 ^de^	6.7	5774.0 ^b^	18.5
1585	*cis*-*β*-Elemene	Ter9	Juice	119.3 ^f^	2.1	133.1 ^ef^	6.7	256.3 ^e^	8.7	123.2 ^f^	1.9	152.1 ^ef^	1.7	228.6 ^ef^	5.9
			Powder	116.1 ^f^	0.4	414.1 ^d^	4.3	1603.4 ^b^	3.1	127.8 ^f^	11	639.0 ^c^	5.9	2011.2 ^a^	6.7
1624	Terpinen-4-ol	Ter10	Juice	30.7 ^f^	12.9	161.2 ^d^	10.4	313.3 ^b^	5.1	38.1 ^ef^	3.3	170.7 ^d^	9.3	345.7 ^b^	9.8
			Powder	6.1 ^f^	3.4	77.8 ^e^	11.6	289.2 ^c^	2.7	22.9 ^f^	7	220.5 ^d^	3.2	414.4 ^a^	8.3
1652	*p*-Menth-1-en-9-al	Ter11	Juice	6.7 ^e^	8.6	50.9 ^d^	19	164.7 ^b^	11	4.3 ^e^	4.5	21.6 ^e^	9.5	175.1 ^b^	11.9
			Powder	3.9^e^	13.8	46.8^d^	13.7	206.4^a^	2.2	2.2^e^	2.0	100.3^c^	9.8	225.0^a^	12.7
1665	*β-Famesene	Ter12	Juice	141.1^c^	12.6	1282.4 ^b^	8.6	5029.2 ^a^	3.1	131.6 ^c^	1.8	164.6 ^c^	3.2	5662.4 ^a^	8.8
			Powder	121.6 ^c^	0.3	1121.4 ^b^	10.2	5795.8 ^a^	9.9	177.9 ^c^	11.4	1467.0 ^b^	16.6	1962.4 ^b^	2.3
1700	*α-Terpineol	Ter13	Juice	24.2 ^g^	17.6	148.9 ^e^	1.6	579.8 ^b^	3.1	53.6 ^fg^	5.9	261.2 ^d^	5.5	712.7 ^a^	10
			Powder	13.2 ^g^	8.2	83.6 ^f^	9.8	376.0 ^c^	2.8	22.4 ^g^	1.4	205.2 ^e^	6.2	568.4 ^b^	3.9
1748	*α-Farnesene	Ter14	Juice	96.8 ^de^	3.8	1698.0 ^c^	14	6332.1 ^a^	1.4	93.7 ^d^	1.7	167.8 ^de^	0.7	6142.2 ^b^	9.4
			Powder	89.1 ^de^	1.8	1355.8 ^c^	11.7	7520.7 ^a^	5.5	129.7 ^d^	2.0	1918.3 ^c^	1.2	6859.0 ^a^	6.6
1754	Geranyl acetate	Ter15	Juice	12.9 ^e^	19.6	220.4 ^de^	14	2135.0 ^b^	4.3	9.2 ^e^	10.5	27.3 ^e^	9.7	2456.8 ^a^	10.1
			Powder	10.9 ^e^	1.0	194.9 ^de^	10.4	1322.0 ^c^	2.5	14.1 ^e^	10.4	435.1 ^d^	9.5	1743.0 ^bc^	5.4
1810	*Nerol	Ter16	Juice	5.8 ^e^	8.5	16.8 ^e^	7.1	146.1 ^b^	2.7	4.1 ^e^	6.9	11.2 ^ef^	14.4	173.6 ^a^	6.6
			Powder	3.6 ^e^	0.6	5.0 ^e^	9.2	56.4 ^d^	8.3	3.8 ^e^	5.3	10.3 ^e^	17.1	77.8 ^c^	12
1814	*cis*-Carveol	Ter17	Juice	4.8 ^de^	0.8	21.5 ^c^	4.5	60.7 ^a^	6.7	2.5 ^de^	11.5	7.2 ^de^	12	59.0 ^a^	8.0
			Powder	0.8 ^e^	2.1	11.3 ^d^	7.8	37.0 ^b^	5.4	1.4 ^de^	21.4	25.2 ^c^	7.7	49.6 ^a^	17.7
1855	*Geraniol	Ter18	Juice	17.1 ^f^	15.1	30.9 ^de^	8.3	116.8 ^a^	4.9	20.7 ^ef^	2.6	27.6 ^de^	6.6	99.5 ^a^	5.2
			Powder	10.7 ^f^	4.3	19.0 ^ef^	9.9	73.9 ^b^	7.8	21.1 ^ef^	5.8	40.4 ^cd^	16.7	44.1 ^c^	12.7
1903	*p*-Menth-1-en-9-ol	Ter19	Juice	N.D.	N/A	4.0 ^d^	7.3	15.1 ^b^	18.5	N.D.	N/A	0.4 ^e^	16	11.8 ^b^	11.2
			Powder	27.8 ^a^	0.3	N.D.	N/A	13.0 ^b^	4.2	N.D.	N/A	N.D.	N/A	9.3 ^c^	14.4
1921	*p*-Mentha-1,8-dien-7-yl acetate	Ter20	Juice	9.2 ^bc^	15.5	23.7 ^bc^	13.4	31.3 ^bc^	0.4	7.5 ^bc^	13	22.0 ^bc^	11.7	22.6 ^bc^	1.0
			Powder	12.0 ^bc^	6.8	24.0 ^bc^	1.8	71.2 ^b^	7.0	28.3 ^bc^	2.0	237.3 ^a^	7.0	N.D.	N/A
1992	Nerolidol	Ter21	Juice	4.1 ^f^	6.2	11.7 ^d^	10.5	31.9 ^b^	14	3.5 ^f^	2.4	4.5 ^f^	15	35.4 ^a^	14.6
			Powder	3.7 ^f^	1.1	8.8 ^ef^	10.4	35.6 ^ab^	6.2	3.5 ^f^	1.9	11.4 ^ef^	12.7	21.4 ^c^	12.3
	**Total**		Juice	***14,336.8***		***13,4467.4***		***830,071***		***13,907.2***		***35,915.9***		***864,557.6***	
			Powder	***12,355.4***		***12,4826.5***		***771,594.3***		***22,672.3***		***285,502.6***		***854,968.4***	
	***Aldehydes***														
1078	*Hexanal	Ald1	Juice	7.3 ^d^	14.4	28.9 ^b^	4.3	55.6 ^a^	12.3	4.5 ^d^	8	7.9 ^d^	8.2	66.4 ^a^	10.5
			Powder	16.1 ^c^	9.4	23.9 ^b^	0.8	21.9 ^bc^	12.9	30.2 ^b^	7.9	7.7 ^d^	12.3	N.D.	N/A
1392	Nonanal	Ald2	Juice	11.5 ^ef^	7.8	20.0 ^de^	7.1	63.5 ^b^	15.9	N.D.	N/A	153.6 ^a^	6.2	29.3 ^d^	12.6
			Powder	14.0 ^ef^	20.5	26.5 ^d^	28.3	53.7 ^c^	13.7	6.6 ^fg^	8.6	27.7 ^d^	9.4	25.2 ^d^	7.7
1474	Decanal	Ald3	Juice	81.5 ^g^	11	894.6 ^de^	0.7	5019.0 ^b^	14.8	83.0 ^g^	9.9	864.7 ^de^	4.5	5356.7 ^b^	15.3
			Powder	197.2 ^fg^	6.5	717.4 ^def^	8.4	3735.8 ^c^	14.4	185.4 ^fg^	4.9	1179.0 ^d^	4.3	9710.3 ^a^	7.2
	**Total**		Juice	***100.3***		***943.5***		***5138.1***		***87.5***		***1026.2***		***5452.3***	
			Powder	***227.3***		***772***		***3811.5***		***222.3***		***1214.3***		***9735.5***	
	***Alcohols***														
1460	1-Heptanol	Alc1	Juice	3.3 ^c^	8.7	3.9 ^c^	7.9	17.9 ^a^	17.4	2.4 ^c^	14.2	3.4 ^c^	20.5	19.8 ^a^	13.3
			Powder	2.6 ^c^	2.9	2.9 ^c^	10.3	7.8 ^b^	9.5	3.4 ^c^	26.8	3.0 ^c^	22.6	4.5 ^c^	12.2
1522	*1-Octanol	Alc2	Juice	20.5 ^fg^	4	64.3 ^e^	5.7	937.7 ^a^	4.1	10.2 ^g^	10.2	125.1 ^d^	23.6	460.9 ^b^	1.8
			Powder	5.1 ^g^	7.1	16.0 ^fg^	15.3	144.1 ^d^	13.2	3.9 ^g^	11.2	43.2 ^ef^	8.9	314.8 ^c^	6.3
	**Total**		Juice	***23.8***		***68.2***		***955.6***		***12.6***		***128.5***		***480.7***	
			Powder	***7.6***		***18.9***		***151.9***		***7.3***		***46.2***		***319.3***	

A1—peel-removed mandarin fruit from Afourer cultivar; A2—flavedo-removed mandarin fruit from Afourer cultivar; A3—whole Afourer mandarin fruit; RS1—peel-removed mandarin fruit from Richard Special cultivar; RS2—flavedo-removed mandarin fruit from Richard Special cultivar; RS3—Whole Richard Special mandarin fruit; Concentrations are presented as average and STD% (standard deviation/average concentration, *n* = 3); different letters (a–g) indicate significant statistical difference (*p* < 0.05) between samples within the same dotted line; ^A^ RI, retention index acquired from the injection of C_7_–C_30_ saturated alkanes under the same chromatographic conditions; ^B^ Compounds with asterisk were identified by comparison of mass spectra and retention time with authentic standards. Compounds without asterisk were identified through the comparison of the obtained mass spectra and retention indices (RIs) with those in the NIST 14 library and NIST Chemistry webbook (https://webbook.nist.gov/chemistry/cas-ser/); ^C^ Compound codes were used in the PCA map; ^D^ ND, not detected in the sample; ^E^ N/A, not applicable.

## Data Availability

Data is contained within the article or Supplementary Material.

## Supplementary Materials

The following are available online at https://www.mdpi.com/2304-8158/10/1/123/s1, Figure S1: Mandarin fruits anatomy. Figure S2: Mandarin juice preparation. Table S1: Calibration curves for the quantification of aroma compounds in mandarin juices and microcapsules. Table S2: Accuracy and precision of the quantification method of phenolic compounds.
