# Supplementary material for: Comparing Three Types of Mandarin Powders Prepared via Microfluidic-Jet Spray Drying: Physical Properties, Phenolic Retention and Volatile Profiling
Source: Foods. 2021 Jan 8;10(1):123. doi: 10.3390/foods10010123 (PMC7827879; doi:10.3390/foods10010123)
Supplement: Supplementary file 1 [file foods-10-00123-s001.pdf]

Supplementary

Comparing Three Types of Mandarin Powders Prepared via Microfluidic-Jet Spray Drying: Physical Properties, Phenolic Retention and Volatile Profiling

Xiao Chen[1], Joanna Le Hoong Ting[1], Yaoyao Peng[1], Pipat Tangjaidee[1], Yongchao Zhu[1], Qili Li[2,3], Yang Shan[2,3*], and Siew Young Quek[1,3,4,*]

[1] Food Science Programme, School of Chemical Sciences, The University of Auckland, Auckland 1010, New Zealand.

[2] Hunan Key Lab of Fruits & Vegetables Storage, Processing, Quality and Safety, Hunan Agricultural Product Processing Institute, Hunan Academy of Agricultural Sciences, Changsha 410125, China

[3] Hunan Province International Joint Lab on Fruits & Vegetables Processing, Quality and Safety, Changsha 410125, China

[4] Riddet Institute, Centre of Research Excellence in Food Research, Palmerston North 4474, New Zealand.

[*] Corresponding author: sy6302@sohu.com (Y.S.); sy.quek@auckland.ac.nz (S.Y.Q)

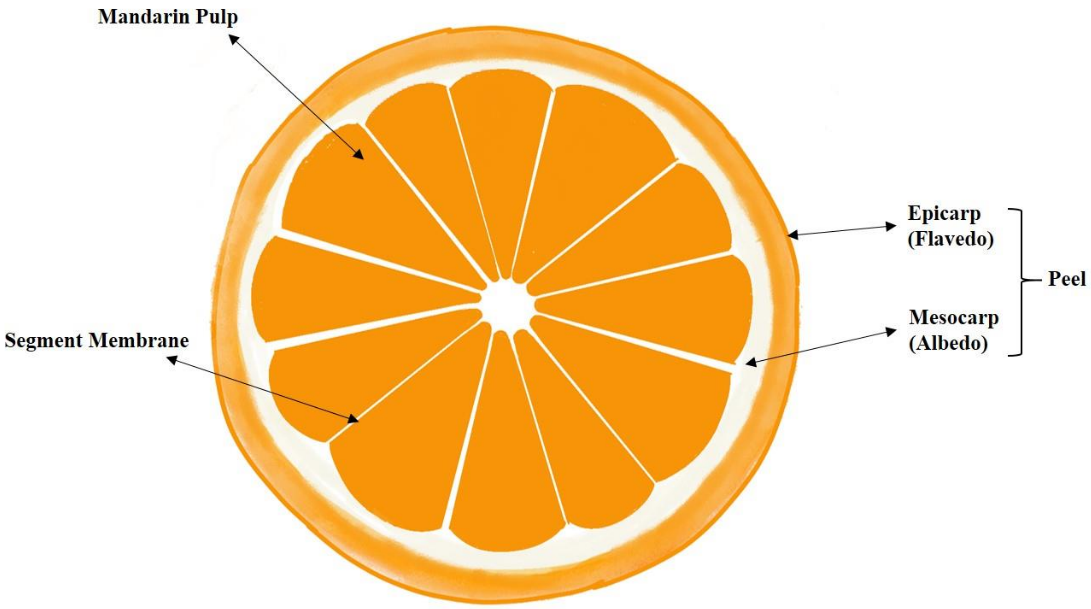

Figure S1. Mandarin fruits anatomy.

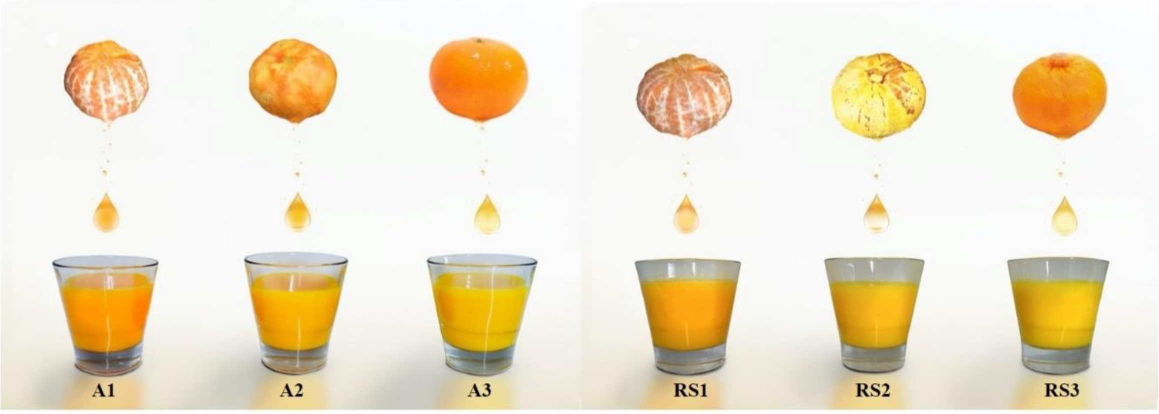

Figure S2. Mandarin juice preparation. A1 – peel-removed mandarin fruit from Afourer cultivar; A2 – flavedo-removed mandarin fruit from Afourer cultivar; A3 – whole Afourer mandarin fruit; RS1 – peel-removed mandarin fruit from Richard Special cultivar; RS2 – flavedo-removed mandarin fruit from Richard Special cultivar; RS3 – Whole Richard Special mandarin fruit.

Table S1. Calibration curves for the quantification of aroma compounds in mandarin juices and microcapsules.

[A]RI	Compound	[B]ION	[C]INSTD	Standard curves	R^2
1018	α-Pinene	93	(±)-linalool-d_3	y=2.0545*x+6.691	0.996
1160	β-Myrcene	93	(±)-linalool-d_3	y=3.3755*x-1.9527	0.999
1180	D-Limonene	68	(±)-linalool-d_3	y=3.4971*x-11.619	0.999
1213	γ-Terpinene	93	(±)-linalool-d_3	y=5.3184*x+13.768	0.998
1700	α-Terpineol	59	α-terpineol-d_3	y=1.2262*x+0.0907	0.996
1665	(*E*)-β-Famesene	93	(±)-linalool-d_3	y=1.4633*x-2.5391	0.976
1810	Nerol	69	(±)-linalool-d_3	y=4.0152*x-0.2087	0.995
1855	Geraniol	69	(±)-linalool-d_3	y=6.1891*x-0.6091	0.999
1078	Hexanal	56	hexanal-d_{12}	y=0.9283*x+0.111	0.996
1522	1-Octanol	56	*n*-hexyl-2,2,3,3,4,4,5,5,6,6,6-d_{11}-alcohol	y=29.064*x-0.0594	0.999

[A] RI, retention index acquired from the injection of C_7-C_{30} saturated alkanes under the same chromatographic conditions as samples; [B] Quantification ion; [C] Internal standard selected for the construction of calibration curves

Table S2. Accuracy and precision of the quantification method of phenolic compounds

	Regression Equation	R^2	Repeatability		Reproducibility		Recovery (%)	LOD (µg/mL)	LOQ (µg/mL)
			Retention time (%RSD)	Peak area (%RSD)	Retention time (%RSD)	Peak area (%RSD)			
**Chlorogenic acid**	y = 30.178x - 17.554	0.9999	0.32	3.23	0.34	3.09	100.27	1.12	3.38
**Hesperidin**	y = 28.019x + 10.088	0.9990	0.08	1.32	0.14	0.87	96.48	0.50	1.51
***p*-coumaric acid**	y = 96.248x + 30.237	0.9991	0.11	0.33	0.18	1.08	94.64	0.38	1.15
***p*-hydroxybenzoic acid**	y = 21.074x + 31.092	0.9959	0.23	3.28	0.26	11.92	120.58	0.80	2.44
**Sinapic acid**	y = 13.995 + 3.5252	0.9991	0.09	1.27	0.17	1.59	95.99	0.36	1.08
**Vanillic acid**	y = 40.111x	0.9974	0.18	14.35	0.18	16.69	90.34	1.30	3.94
